# Relative validity of bioelectrical impedance analysis in estimating body composition in women with overweight and obesity 2 weeks and 6 months postpartum

**DOI:** 10.29219/fnr.v69.10869

**Published:** 2025-01-24

**Authors:** Elin Westerheim, Elisabeth A. Øhman, Maria Fossli, Anna Winkvist, Hege Berg Henriksen, Hilde K. Brekke

**Affiliations:** 1 Department of Nutrition, Institute of Basic Medical Sciences, University of Oslo, Norway; 2 Norwegian Research Centre for Women’s Health, Oslo University Hospital, Oslo, Norway; 3 Department of Internal Medicine and Clinical Nutrition, Sahlgrenska Academy, University of Gothenburg, Gothenburg, Sweden

**Keywords:** bioelectrical impedance, fat mass, fat free mass, DXA, overweight, postpartum

## Abstract

**Objective:**

To investigate the relative validity of bioelectrical impedance analysis (BIA) in estimating fat mass (FM) and fat free mass (FFM) with dual-energy X-ray absorptiometry (DXA) as reference method in women with overweight and obesity 2 weeks and 6 months postpartum (pp).

**Methods:**

Body composition of 94 women with overweight and obesity was assessed using Seca mBCA 515 and GE Healthcare Lunar iDXA. Agreement between the two methods for FM and FFM at 2 weeks and 6 months pp, as well as the changes in FM and FFM between the two timepoints, were tested using paired *t*-test, Bland–Altman plots and regression analyses.

**Results:**

The mean (standard deviation [SD]) body mass index (BMI) at 2 weeks pp was 30.6 (2.6) kg/m^2^ and mean (SD) weight loss at 6 months pp was 4.7 (4.8) kg. BIA underestimated FM at both 2 weeks pp and 6 months pp by mean (SD) 0.7 (1.4) kg and 0.3 (1.3) kg and overestimated FFM at both timepoints by 1.2 (1.5) kg and 0.7 (1.4) kg, with proportional bias for FFM. BIA underestimated *changes* in FM by mean (SD) 0.5 (1.1) kg and overestimated *changes* in FFM by 0.5 (1.0) kg, with proportional bias for change in extracellular water by total body water. Agreement was generally high for both cross-sectional and longitudinal comparisons.

**Conclusions:**

At group level, BIA was a valid tool for assessment of FM and FFM in women with overweight and obesity at 2 weeks and 6 months pp when compared to DXA. We also consider it valid for following changes in FM and FFM over time when fluid distribution is stable.

## Popular scientific summary

- This study compared BIA to DXA as a method for measuring fat mass and fat-free mass in women with overweight and obesity at 2 weeks and 6 months postpartum, and also examined BIA’s ability to measure changes in body composition between these time points.
- At group level, BIA was a valid tool for assessing fat mass and fat free mass compared to DXA.
- BIA was valid for assessing changes in body composition over time when fluid distribution was stable.

Childbearing is a risk factor for long term weight gain (1, 2). Optimizing weight management after pregnancy, by decreasing fat mass (FM) and preserving fat-free mass (FFM), may reduce both long-term risks of obesity-related disorders, as well as risk of obesity-related obstetric complications in future pregnancies (3).

Affordable, easily accessible and valid equipment to assess body composition and to monitor changes in body composition in postpartum (pp) women with overweight and obesity are needed. Equipment that has been validated for normal weight, non-pregnant, non-lactating women are not necessarily valid in this group.

Bioelectrical impedance analysis (BIA) is widely used in the clinic and assumes that the body consists of two compartments only; FM and FFM, but has several advantages. The equipment is safe, the measurement is simple and non-invasive, and the results are reproducible and easily obtained (4). There are different BIA devices available, from simple single frequency devices to multi-frequency devices. Whole body BIA devices consider the body as one single, conducting cylinder, while segmental BIA devices consider the body as five conducting cylinders, that is, two legs, to arms and the trunk. A segmental, multi frequency BIA device is anticipated to be more reliable than simpler devices (5). When using appropriate population, age and pathology-specific BIA equations, BIA allows the determination of total body water (TBW), and thereby also FFM and FM, in subjects without significant fluid and electrolyte abnormalities (4).

Dual energy X-ray absorptiometry (DXA) has become an attractive technique for obtaining valid measurements of FM and lean mass. Different types of BIA have been validated against several other methods, including DXA. BIA has been found to significantly underestimate the amount of FM (6–11) and overestimate the amount of FFM (5, 9–13). Body composition estimates by BIA are less accurate in overweight and obese subjects, compared to normal-weight subjects (12). Kyle et al. (14) concluded that BIA is valid up to a body mass index (BMI) of 34 kg/m^2^ and must be interpreted with caution in subjects with a BMI > 34 kg/m^2^ (15, 16). For the BIA, equations have been developed especially for obese subjects (16).

Pregnancy causes dramatic changes in body composition. Fluid retention is a common feature (17). Measuring body composition by BIA in women with higher BMIs and fluid retention from pregnancy thus poses an extra challenge that needs to be addressed. BIA has been found to be suitable to assess postpartum body composition at 1 and 4 months pp, although in a small sample of women with a wide range in BMI (11).

The aim of this research is to investigate the validity of BIA in estimating FM and FFM as well as changes in FM and FFM over time, with DXA as reference method, in women with overweight and obesity at 2 weeks and 6 months pp.

## Methods

### Participants and study design

The study participants were taking part in a larger study on weight loss and breastfeeding pp. Through online advertisements, 156 women in the Oslo area were recruited to the main study. They were pregnant women with a self-reported, pre-pregnancy BMI of 25–35 kg/m^2^, able to read and write Norwegian, with an intention to breastfeed, no previous breast reduction surgery and singleton pregnancy. Chronic diseases or medications known to affect lipid and glucose metabolism, development of pre-eclampsia or drug-treated gestational diabetes, abortion or still birth or short gestation (<36 weeks) and new pregnancy were exclusion criteria.

In the main study, participants were randomized to either breastfeeding promotion intervention (BPI) or diet intervention in a 2 × 2 factorial design. Of the 156 participants recruited between 30 January 2018 and 3 December 2021, 94 participants who had performed both BIA and DXA scans at 2 weeks and 6 months pp, were included in the present validation study. At the first visit, 55 women (58.5%) had received BPI and at 6 months pp the distribution between the groups were: BPI: 29 women, BPI & diet: 26 women, diet: 18 women, No treatment: 21 women. The diet intervention had a weight loss goal of 6 kg from 10 to 22 weeks pp. Effects of treatments are not evaluated here, only associations related to changes in BMI and distribution of body water.

Anthropometric measurement and a single body composition assessment using BIA and DXA were conducted after an overnight fast. However, since the participants were breastfeeding, they were allowed to drink up to 200 mL water between midnight and measurement.

The main study EVA (in Norwegian: Effekter av Vektnedgang og Amming [Effects of Weight loss and Lactation], ClinicalTrials no NCT03580057) was approved by Regional Ethics Committee (2017/451) and all participants consented to take part in the study before any measurements were performed. The study was conducted at the Department of Nutrition, University of Oslo, Norway.

### Body composition measurements

#### Seca mBCA 515

Height was measured once at 2 weeks pp, and measured to the closest 0.5 cm with a wall-mounted digital display stadiometer (Seca 264, Hamburg, Germany). Weight was measured on the BIA device (Seca mBCA 515, Hamburg, Germany), in connection to impedance measurement, and registered to the closest 0.1 kg. The participants were barefoot and wearing light clothing. When the device obtained contact with both feet and hands, the impedance was measured automatically (single measurement). In addition to FM and FFM from the manufacturer’s in-built prediction equations, data on TBW, extracellular water (ECW) and ratio of ECW to TBW were retrieved. Waist circumference was measured at the midpoint of the lower margin of the last palpable rib and the top of the iliac crest to the nearest 0.5 cm using a measuring tape. Hip circumference was measured around the widest part of the hips.

#### Lunar iDXA

We used the Lunar iDXA (GE Healthcare Lunar, Buckinghamshire, United Kingdom) at the Department of Nutrition, University of Oslo, Norway. This Lunar iDXA device has shown high precision and valid measurements of body compartments (18, 19). Daily calibrations and measurements were performed by trained personnel and according to standard operation procedure, including removal of jewelry, the use of lightweight clothing, and in standardized positioning (20).

For participants exceeding the scan field, the composition of the body part exceeding the scan field on the right side was estimated based on the information from the left side, anticipating symmetry of the body. After completing the scanning, the lines for Region of Interest (ROI) were adjusted and the images were checked for completeness and quality. The head line was placed just below the lower boundary of the chin bone and the pelvis line was placed just above the upper boundaries of the iliac crests. In some cases, also other ROI lines were adjusted, in order to obtain as correct analysis of the body composition as possible, according to the standard procedures (20).

### Statistical analyses

Data analyses were performed with SPSS statistics software, IBM SPSS Statistics for Windows, version 28.0.0.0 (IBM Corp., Armonk, N.Y., USA). Descriptive statistics are presented as means and standard deviation (SD). Anthropometric variables, DXA and BIA values, the differences in FM and FFM between DXA and BIA, as well as the calculated changes in different variables between the two visits, were normally distributed.

Paired-samples *t*-test was used to compare DXA and BIA at 2 weeks and 6 months pp, and to compare the difference in change from 2 weeks to 6 months between the methods. Pearson correlations were used to illustrate associations between the two methods. Bland–Altman’s scatter plots were used to illustrate agreement, systematic and proportional bias between DXA and BIA, that is, BIA as test method against DXA as reference method, in estimating FM and FFM at 2 weeks and 6 months pp. Possible bias by BMI and distribution of body water, measured as percent ECW of TBW, as well as changes in these variables between 2 weeks and 6 months, were also examined using scatter plots and linear regression analysis.

## Results

The mean (SD) age was 33.2 (3.7) years (Table 1). Almost half were primi parous and the vast majority (99%) were breastfeeding. Mean (SD) BMI at 2 weeks pp was 30.6 (2.6) kg/m^2^ (range 26.1–37.7) (Table 2). The mean FM constituted 41% of the body weight at 2 weeks pp estimated by BIA and 42% by DXA.

**Table 1:** Main characteristics of the 94 women with a pre-pregnancy BMI of 25–35 kg/m^2^ completing baseline visit at 2 weeks postpartum and follow-up visit 6 months postpartum

	*N*	Mean (SD)	%
**Age (years)**	94	33.2 (3.7)	
**Height (m)**	94	1.68 (0.06)	
**Parity (previous)**			
0	45		47.9
1	43		45.7
2	6		6.4
**Pre pregnancy BMI**	94	28.8 (2.6)	
**Duration of pregnancy (weeks)**	94	40.1 (1.2)	
**Days between partum and baseline**	94	15.1 (5.0)	
**Weeks between partum and follow-up**	48	24.4 (2.6)	
**Type of delivery**			
Vaginal	76		80.9
Caesarean	18		19.1
**Breastfeeding at 2 weeks postpartum**			
Full breastfeeding	81		86
Partial breastfeeding	12		13
No breastfeeding	1		1
**Breastfeeding at 6 months postpartum**			
Full breastfeeding	15		16
Partial breastfeeding	76		81
No breastfeeding	3		3

**Table 2:** Anthropometric variables and body composition variables from DXA and BIA examinations of the 94 women with a pre-pregnancy BMI of 25–35 kg/m^2^ completing the baseline visit at 2 weeks postpartum and the follow-up visit at 6 months postpartum

	Baseline visit at 2 weeks postpartum		Follow-up visit at 6 months postpartum	Change baseline visit – follow-up visit	*P* forchange
	*n*	Mean (SD)	Mean (SD)	Mean (SD)	
**Weight (kg)**	94	87.0 (9.3)	82.3 (10.7)	−4.7 (4.8)	<0.001
**BMI (kg/m^2^)**	94	30.6 (2.6)	29.0 (3.1)	−1.7 (1.7)	<0.001
**Waist circ. (cm)**	94	98.9 (8.4)	92.0 (9.1)	−6.9 (7.1)	<0.001
**Hip circ. (cm)**	94	115.9 (6.8)	111.1 (7.6)	−4.8 (4.2)	<0.001
**FM (kg)**					
DXA	94	36.5 (6.3)	33.6 (7.7)	−2.9 (4.1)	<0.001
BIA Difference DXA-BIA	94 94	35.7 (6.2) 0.7 (1.4)	33.3 (7.6) 0.3 (1.3)	−2.4 (4.1) 0.5 (1.1)	<0.001 <0.001
**FFM (kg)**					
DXA	94	50.1 (5.1)	48.3 (5.0)	−1.8 (1.7)	<0.001
BIA Difference DXA-BIA	94 94	51.2 (4.6) −1.2 (1.5)	49.0 (4.5) −0.7 (1.4)	−2.3 (1.5) −0.5 (1.0)	<0.001 <0.001
**BIA variables**					
TBW (kg)	94	38.2 (3.5)	36.4 (3.5)	−1.8 (1.2)	<0.001
ECW (kg)	94	17.1 (1.6)	16.2 (1.6)	−0.9 (0.7)	<0.001
ECW:TBW (%)	94	44.8 (1.3)	44.4 (1.2)	−0.4 (1.0)	0.001

DXA, dual energy X-ray absorptiometry; BIA, bioelectrical impedance analysis; FM, fat mass; FFM, fat free mass; TBW, total body water; ECW, extracellular water

### Cross-sectional validation of BIA AGAINST DXA at 2 weeks and at 6 months pp

Pearson correlation test showed a high and significant correlation between BIA and DXA in measuring FM and FFM at both visits (*r* = 0.96–0.99, all *P* < 0.001).

BIA underestimated FM by mean (SD) 0.7 (1.4) kg (2.0%) (*P* < 0.001) at 2 weeks pp and 0.3 (1.3) kg (0.7%) (*P* = 0.038) at the visit 6 months pp, while it overestimated FFM by 1.2 (1.5) kg (2.5%) (*P* < 0.001) at 2 weeks pp and 0.7 (1.4) kg (1.5%) (*P* < 0.001) at 6 months pp.

**Fig. 1:**
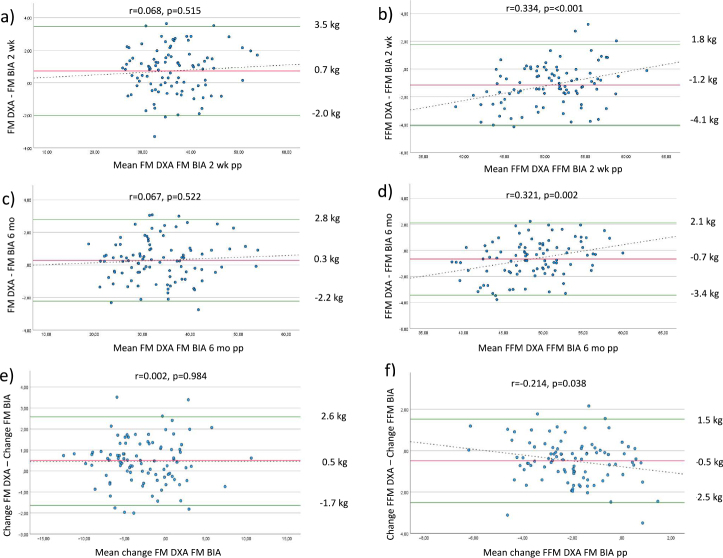
Bland-Altman plots of cross-sectional comparison between DXA and BIA at 2 weeks (a, b) and 6 months (c, d) as well as longitudinal change between 2 weeks and 6 months (e, f) postpartum. DXA, dual energy X-ray absorptiometry; BIA, bioelectrical impedance analysis; FM, fat mass; FFM, fat free mass; pp, postpartum; wk, weeks; mo, months. y-axis shows reference method (DXA) minus test method (BIA).

For FM limits of agreement at 2 weeks ranged from −2.0 to 3.5 kg (Fig. 1a) and at 6 months from −2.2 to 2.8 kg (Fig. 1c). For FFM limits of agreement at 2 weeks ranged from −4.1 to 1.8 kg (Fig. 1b) and at 6 months from −3.4 to 2.1 kg (Fig. 1d).

Regression of the difference between the two methods against the mean of the two methods for FFM was significant at 2 weeks and 6 months, both *P* < 0.005 (Fig. 1b and 1d).

BMI and distribution of body water, measured as ECW/TBW, showed no association with FM or FFM at 2 weeks or 6 months (Supplementary Fig. 1a to h).

### Ability of BIA to measure changes in body composition compared to DXA

The participants decreased in total weight and waist and hip circumference from 2 weeks pp to 6 months pp, along with a decrease in FM and FFM measured by both DXA and BIA, as well as a decrease in ECW/TBW (Table 2).

Pearson correlation for changes in FM and FFM between the two visits measured by DXA and BIA were 0.97 and 0.81 (*P* < 0.001), respectively.

Compared to DXA, BIA underestimated the change in FM by 0.5 (1.1) kg (*P* < 0.001) and overestimated the change in FFM by 0.5 (1.0) kg (*P* < 0.001), (Table 2).

Limits of agreement for changes in FM and FFM ranged from −1.7 to 2.6 kg and −2.5 to 1.5 kg, respectively (Fig. 1e and f).

For FM, there was no proportional bias between the methods, while for FFM there was a small proportional bias (Fig. 1f).

**Fig. 2:**
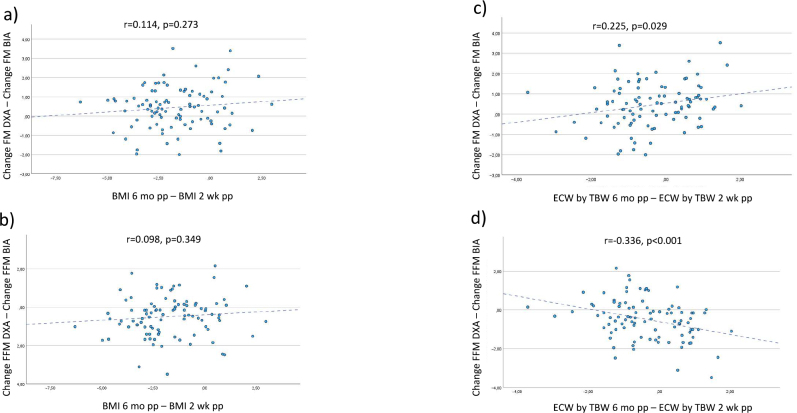
Plots of the difference in change between FM and FFM by DXA – BIA and difference in BMI (a, b) as well as difference in ECW by TBW (c, d) between 2 weeks and 6 months postpartum. DXA, dual energy X-ray absorptiometry; BIA, bioelectrical impedance analysis; FM, fat mass; FFM, fat free mass; pp, postpartum; wk, weeks; mo, months. y-axis shows reference method (DXA) minus test method (BIA).

There was no proportional bias for change in BMI (Fig. 2a and b), however, regression of the difference in change between the two methods against change in ECW/TBW was significant for both FM and FFM, *P* = 0.029 and *P* < 0.001, respectively, (Fig. 2c and d).

## Discussion

Similar to previous studies comparing BIA with DXA in cross-sectional samples (5–13), we also found in this sample of women with overweight or obesity that BIA underestimated the amount of FM and overestimated the amount of FFM compared to DXA, however, with narrower limits of agreement. This applied to measurements at both 2 weeks and 6 months pp. There was a proportional bias for overestimating FFM in the lower FFM range. On a group level, BIA underestimated the change in FM and overestimated the change in FFM from 2 weeks to 6 months pp, compared to DXA. The overestimation of change in FFM was larger in those women who increased their ECW relative to TBW.

### Cross-sectional comparison at 2 weeks and 6 months

The difference between the methods in measuring FM and FFM was larger at 2 weeks pp (2.0–2.5%) compared to 6 months pp (0.7–1.5%). Similarly, limits of agreement were wider at 2 weeks pp (±2.7 – ±2.9) compared to 6 months pp (±2.5 – ±2.8). Thus, it seems that BIA has better precision in estimating both FM and FFM at 6 months pp. Considering the expected ‘normalization’ in hydration over time pp, this is not surprising (21). However, there was no proportional bias by the ratio of extracellular water to TBW at the two timepoints.

The limits of agreement seen here were more narrow, actually halved, compared to what we have shown in a previous study in a similar population of pp overweight and obese women in Sweden, using bioelectrical impedance spectroscopy (BIS) compared to DXA (8). The observed limits of agreement in this study are more in line with a study that used the same two devices with manufacturers’ equations in pp women at 1 and 4 months pp (11), indicating that this eight-electrode BIA has high relative validity in comparison to earlier generations of BIA devices. Although mean bias for FFM in pp women may be somewhat larger in comparison to studies using the same equipment in healthy men and women (9) and colorectal cancer patients (22), limits of agreement are generally more narrow. One likely explanation is the homogeneity of our population in terms of gender, age and BMI.

### Measuring longitudinal changes in body composition

We found that BIA underestimated the change in FM by mean (SD) 0.5 (1.1) kg and overestimated the change in FFM by 0.5 (1.0) kg, which is similar to the results of Verdich et al. (23) who compared multifrequency BIA to Lunar iDXA during weigh loss. However, Bärebring et al. (24) found that single frequency BIA underestimated change in FFM during follow-up in colorectal patients. Differences between studies may be due to differences in weight change, population and type of BIA equipment.

The limits of agreement were approximately ±2 kg for longitudinal changes in both FM and FFM. This is about half of what Verdich et al. and Bärebring et al. found in their studies (23, 24). It is also much more narrow than we previously showed when comparing BIS to DXA (Lunar Prodigy) in a similar group of women (8). Thus, it seems that the Seca mBCA 515 has better agreement compared to other BIA devices, also when it comes to measuring longitudinal changes.

The ratio of ECW/TBW decreased significantly between the two timepoints and there was a highly significant proportional bias for measuring change in FM and FFM by change in water retention. Hopkinson et al. have shown that hydration of FFM in women 2 weeks pp is increased (0.75) compared to ‘normal’ hydration (0.73, typically assumed in 2-compartment models) and that hydration was higher in lactating (0.75) compared to non-lactating (0.74) women (25). These pregnancy-induced changes in hydration were no longer evident at 3 months pp, nor were there any differences between lactating and non-lactating women at that timepoint (21). Thus, although the manufacturers equation of this BIA device does not show any proportional bias with water retention in the cross-sectional comparison with DXA, the change in hydration seems to affect the agreement between BIA and DXA when measuring longitudinal change.

GE Healthcare Lunar iDXA has a precision error found to be small, less than 1% (26), also for the actual machine used in our study (19). It is important to notice that DXA also assumes a fixed hydration of 73% (27) while the four-compartment model, which is considered the gold standard for measuring body composition (28–30), does not assume a fixed hydration. Thus, DXA has some of the same limitations as BIA and may be affected by the hydration status of our participants. Although it has been estimated that fat free tissue hydration between 68.2 and 78.2% does not significantly alter total percentage of fat (31), it has been shown that diet and training regimens that induce short-term changes in hydration can manipulate lean tissue mass results (32). The data on pp women from Butte et al. indicate that the composition of FFM in our participants are not affected by their pp or lactation status at 6 months pp as composition of FFM is expected to be normalized by 3 months pp (21). However, our results at 2 weeks pp may well be affected.

Although previous researchers have concluded that BIA is not a reliable method to track changes in body composition (13, 33), especially not in an overweight population (33), our results showed a good correlation between Seca mBCA 515 and DXA, with a degree of underestimation of changes in FM and overestimation of changes in FFM that for most purposes could be considered acceptable in a clinical setting. Garr Barry et al. (11) did also find the same BIA to be reliable for measuring FM and FFM in women at 1 and 4 months pp, both cross sectionally and longitudinally, in a similar, but smaller group of women, although weight stable. In addition, we have shown that this applies to women already 2 weeks pp and until 6 months pp, also during weight loss and without proportional bias for change in BMI, but with a proportional bias for change in hydration.

### Limitations

The measurements in this comparison study were performed over a period of 4 years by several members of the project team and an interobserver variation cannot be excluded. However, training and standard operation procedures have been in place in order to minimize the variation.

The measurements were taken between 8 o’clock and 11 o’clock in the morning and the duration in an upright position may have affected BIA measurement (34). Neither intake of water (up to 200 mL) or time since last breastfeeding were recorded.

The light clothing was not standardized and was thus included in the calculations of FM and FFM made by BIA, but not by DXA.

It is unlikely that the diet intervention or BPI would affect the difference between the two methods, cross-sectionally or longitudinally, although this possibility cannot be excluded. Most women lose weight naturally from 2 weeks to 6 months pp and in addition half of the participants received weight loss treatment. Therefore, the results obtained here are mainly limited to a population undergoing weight loss pp.

## Conclusion

At group level, BIA was a valid tool compared to DXA for assessment of FM and FFM in women with overweight and obesity at 2 weeks and 6 months pp although the agreement improved with time pp. We also considered it valid for following changes in FM and FFM over time when fluid distribution is stable.

## Supplementary Material



## Data Availability

The datasets generated during and/or analyzed during this study are not publicly available due to risk of re-identifying participants but are available from the corresponding author on reasonable request.
