# Supplementary material for: Relative validity of bioelectrical impedance analysis in estimating body composition in women with overweight and obesity 2 weeks and 6 months postpartum
Source: Food Nutr Res. 2025 Jan 24;69:10.29219/fnr.v69.10869. doi: 10.29219/fnr.v69.10869 (PMC11836777; doi:10.29219/fnr.v69.10869)
Supplement: Supplementary file 1 [file FNR-69-10869-s1.pdf]

food & nutrition research

ORIGINAL ARTICLE

Relative validity of bioelectrical impedance analysis in estimating body composition in women with overweight and obesity 2 weeks and 6 months postpartum

Elin Westerheim[1], Elisabeth A. Øhman[1], Maria Fossli[1,2], Anna Winkvist[3], Hege Berg Henriksen[1] and Hilde K. Brekke[1]*

[1]Department of Nutrition, Institute of Basic Medical Sciences, University of Oslo, Norway; [2]Norwegian Research Centre for Women's Health, Oslo University Hospital, Oslo, Norway; [3]Department of Internal Medicine and Clinical Nutrition, Sahlgrenska Academy, University of Gothenburg, Gothenburg, Sweden

Popular scientific summary

- This study compared BIA to DXA as a method for measuring fat mass and fat-free mass in women with overweight and obesity at 2 weeks and 6 months postpartum, and also examined BIA's ability to measure changes in body composition between these time points.
- At group level, BIA was a valid tool for assessing fat mass and fat free mass compared to DXA.
- BIA was valid for assessing changes in body composition over time when fluid distribution was stable.

Abstract

Objective: To investigate the relative validity of bioelectrical impedance analysis (BIA) in estimating fat mass (FM) and fat free mass (FFM) with dual-energy X-ray absorptiometry (DXA) as reference method in women with overweight and obesity 2 weeks and 6 months postpartum (pp).
Methods: Body composition of 94 women with overweight and obesity was assessed using Seca mBCA 515 and GE Healthcare Lunar iDXA. Agreement between the two methods for FM and FFM at 2 weeks and 6 months pp, as well as the changes in FM and FFM between the two timepoints, were tested using paired *t*-test, Bland–Altman plots and regression analyses.
Results: The mean (standard deviation [SD]) body mass index (BMI) at 2 weeks pp was 30.6 (2.6) kg/m^2 and mean (SD) weight loss at 6 months pp was 4.7 (4.8) kg. BIA underestimated FM at both 2 weeks pp and 6 months pp by mean (SD) 0.7 (1.4) kg and 0.3 (1.3) kg and overestimated FFM at both timepoints by 1.2 (1.5) kg and 0.7 (1.4) kg, with proportional bias for FFM. BIA underestimated *changes* in FM by mean (SD) 0.5 (1.1) kg and overestimated *changes* in FFM by 0.5 (1.0) kg, with proportional bias for change in extracellular water by total body water. Agreement was generally high for both cross-sectional and longitudinal comparisons.
Conclusions: At group level, BIA was a valid tool for assessment of FM and FFM in women with overweight and obesity at 2 weeks and 6 months pp when compared to DXA. We also consider it valid for following changes in FM and FFM over time when fluid distribution is stable.

Keywords: *bioelectrical impedance*; *fat mass*; *fat free mass*; *DXA*; *overweight*; *postpartum*

To access the supplementary material, please visit the article landing page

Received: 26 June 2024; Revised: 9 December 2024; Accepted: 18 December 2024; Published: 24 January 2025

Childbearing is a risk factor for long term weight gain (1, 2). Optimizing weight management after pregnancy, by decreasing fat mass (FM) and preserving fat-free mass (FFM), may reduce both long-term risks of obesity-related disorders, as well as risk of obesity-related obstetric complications in future pregnancies (3).

Affordable, easily accessible and valid equipment to assess body composition and to monitor changes in body composition in postpartum (pp) women with overweight and obesity are needed. Equipment that has been validated for normal weight, non-pregnant, non-lactating women are not necessarily valid in this group.

Food & Nutrition Research 2025. © 2025 Elin Westerheim et al. This is an Open Access article distributed under the terms of the Creative Commons Attribution 4.0 International License (http://creativecommons.org/licenses/by/4.0/), allowing third parties to copy and redistribute the material in any medium or format and to remix, transform, and build upon the material for any purpose, even commercially, provided the original work is properly cited and states its license.

Bioelectrical impedance analysis (BIA) is widely used in the clinic and assumes that the body consists of two compartments only; FM and FFM, but has several advantages. The equipment is safe, the measurement is simple and noninvasive, and the results are reproducible and easily obtained (4). There are different BIA devices available, from simple single frequency devices to multi-frequency devices. Whole body BIA devices consider the body as one single, conducting cylinder, while segmental BIA devices consider the body as five conducting cylinders, that is, two legs, to arms and the trunk. [AQ3] A segmental, multi frequency BIA device is anticipated to be more reliable than simpler devices (5). When using appropriate population, age and pathology-specific BIA equations, BIA allows the determination of total body water (TBW), and thereby also FFM and FM, in subjects without significant fluid and electrolyte abnormalities (4).

Dual energy X-ray absorptiometry (DXA) has become an attractive technique for obtaining valid measurements of FM and lean mass. Different types of BIA have been validated against several other methods, including DXA. BIA has been found to significantly underestimate the amount of FM (6–11) and overestimate the amount of FFM (5, 9–13). Body composition estimates by BIA are less accurate in overweight and obese subjects, compared to normal-weight subjects (12). Kyle et al. (14) concluded that BIA is valid up to a body mass index (BMI) of 34 kg/m^2 and must be interpreted with caution in subjects with a BMI > 34 kg/m^2 (15, 16). For the BIA, equations have been developed especially for obese subjects (16).

Pregnancy causes dramatic changes in body composition. Fluid retention is a common feature (17). Measuring body composition by BIA in women with higher BMIs and fluid retention from pregnancy thus poses an extra challenge that needs to be addressed. BIA has been found to be suitable to assess postpartum body composition at 1 and 4 months pp, although in a small sample of women with a wide range in BMI (11).

The aim of this research is to investigate the validity of BIA in estimating FM and FFM as well as changes in FM and FFM over time, with DXA as reference method, in women with overweight and obesity at 2 weeks and 6 months pp.

Methods

Participants and study design

The study participants were taking part in a larger study on weight loss and breastfeeding pp. Through online advertisements, 156 women in the Oslo area were recruited to the main study. They were pregnant women with a self-reported, pre-pregnancy BMI of 25–35 kg/m^2, able to read and write Norwegian, with an intention to breastfeed, no previous breast reduction surgery and singleton pregnancy. Chronic diseases or medications known to affect lipid and glucose metabolism, development of pre-eclampsia or drug-treated gestational diabetes, abortion or still birth or short gestation (<36 weeks) and new pregnancy were exclusion criteria.

In the main study, participants were randomized to either breastfeeding promotion intervention (BPI) or diet intervention in a 2 × 2 factorial design. Of the 156 participants recruited between 30 January 2018 and 3 December 2021, 94 participants who had performed both BIA and DXA scans at 2 weeks and 6 months pp, were included in the present validation study. At the first visit, 55 women (58.5%) had received BPI and at 6 months pp the distribution between the groups were: BPI: 29 women, BPI & diet: 26 women, diet: 18 women, No treatment: 21 women. The diet intervention had a weight loss goal of 6 kg from 10 to 22 weeks pp. Effects of treatments are not evaluated here, only associations related to changes in BMI and distribution of body water.

Anthropometric measurement and a single body composition assessment using BIA and DXA were conducted after an overnight fast. However, since the participants were breastfeeding, they were allowed to drink up to 200 mL water between midnight and measurement.

The main study EVA (in Norwegian: Effekter av Vektnedgang og Amming [Effects of Weight loss and Lactation], ClinicalTrials no NCT03580057) was approved by Regional Ethics Committee (2017/451) and all participants consented to take part in the study before any measurements were performed. The study was conducted at the Department of Nutrition, University of Oslo, Norway.

Body composition measurements

Seca mBCA 515

Height was measured once at 2 weeks pp, and measured to the closest 0.5 cm with a wall-mounted digital display stadiometer (Seca 264, Hamburg, Germany). Weight was measured on the BIA device (Seca mBCA 515, Hamburg, Germany), in connection to impedance measurement, and registered to the closest 0.1 kg. The participants were barefoot and wearing light clothing. When the device obtained contact with both feet and hands, the impedance was measured automatically (single measurement). In addition to FM and FFM from the manufacturer's in-built prediction equations, data on TBW, extracellular water (ECW) and ratio of ECW to TBW were retrieved. Waist circumference was measured at the midpoint of the lower margin of the last palpable rib and the top of the iliac crest to the nearest 0.5 cm using a measuring tape. Hip circumference was measured around the widest part of the hips.

Lunar iDXA

We used the Lunar iDXA (GE Healthcare Lunar, Buckinghamshire, United Kingdom) at the Department of Nutrition, University of Oslo, Norway. This Lunar iDXA device has shown high precision and valid measurements

Citation: Food & Nutrition Research 2025, 69: 10869 - http://dx.doi.org/10.29219/fnr.v69.10869

of body compartments (18, 19). Daily calibrations and measurements were performed by trained personnel and according to standard operation procedure, including removal of jewelry, the use of lightweight clothing, and in standardized positioning (20).

For participants exceeding the scan field, the composition of the body part exceeding the scan field on the right side was estimated based on the information from the left side, anticipating symmetry of the body. After completing the scanning, the lines for Region of Interest (ROI) were adjusted and the images were checked for completeness and quality. The head line was placed just below the lower boundary of the chin bone and the pelvis line was placed just above the upper boundaries of the iliac crests. In some cases, also other ROI lines were adjusted, in order to obtain as correct analysis of the body composition as possible, according to the standard procedures (20).

Statistical analyses

Data analyses were performed with SPSS statistics software, IBM SPSS Statistics for Windows, version 28.0.0.0 (IBM Corp., Armonk, N.Y., USA). Descriptive statistics are presented as means and standard deviation (SD). Anthropometric variables, DXA and BIA values, the differences in FM and FFM between DXA and BIA, as well as the calculated changes in different variables between the two visits, were normally distributed.

Paired-samples *t*-test was used to compare DXA and BIA at 2 weeks and 6 months pp, and to compare the difference in change from 2 weeks to 6 months between the methods. Pearson correlations were used to illustrate associations between the two methods. Bland–Altman's scatter plots were used to illustrate agreement, systematic and proportional bias between DXA and BIA, that is, BIA as test method against DXA as reference method, in estimating FM and FFM at 2 weeks and 6 months pp. Possible bias by BMI and distribution of body water, measured as percent ECW of TBW, as well as changes in these variables between 2 weeks and 6 months, were also examined using scatter plots and linear regression analysis.

Results

The mean (SD) age was 33.2 (3.7) years (Table 1). Almost half were primi parous and the vast majority (99%) were breastfeeding. Mean (SD) BMI at 2 weeks pp was 30.6 (2.6) kg/m^2 (range 26.1–37.7) (Table 2). The mean FM constituted 41% of the body weight at 2 weeks pp estimated by BIA and 42% by DXA.

Cross-sectional validation of BIA AGAINST DXA at 2 weeks and at 6 months pp

Pearson correlation test showed a high and significant correlation between BIA and DXA in measuring FM and FFM at both visits (r = 0.96–0.99, all $P < 0.001$).

Table 1. Main characteristics of the 94 women with a pre-pregnancy BMI of 25–35 kg/m^2 completing baseline visit at 2 weeks postpartum and follow-up visit 6 months postpartum

	N	Mean (SD)	%
**Age (years)**	94	33.2 (3.7)	
**Height (m)**	94	1.68 (0.06)	
**Parity (previous)**			
0	45		47.9
1	43		45.7
2	6		6.4
**Pre pregnancy BMI**	94	28.8 (2.6)	
**Duration of pregnancy (weeks)**	94	40.1 (1.2)	
**Days between partum and baseline**	94	15.1 (5.0)	
**Weeks between partum and follow-up**	48	24.4 (2.6)	
**Type of delivery**			
Vaginal	76		80.9
Caesarean	18		19.1
**Breastfeeding at 2 weeks postpartum**			
Full breastfeeding	81		86
Partial breastfeeding	12		13
No breastfeeding	1		1
**Breastfeeding at 6 months postpartum**			
Full breastfeeding	15		16
Partial breastfeeding	76		81
No breastfeeding	3		3

BIA underestimated FM by mean (SD) 0.7 (1.4) kg (2.0%) ($P < 0.001$) at 2 weeks pp and 0.3 (1.3) kg (0.7%) ($P = 0.038$) at the visit 6 months pp, while it overestimated FFM by 1.2 (1.5) kg (2.5%) ($P < 0.001$) at 2 weeks pp and 0.7 (1.4) kg (1.5%) ($P < 0.001$) at 6 months pp.

For FM limits of agreement at 2 weeks ranged from −2.0 to 3.5 kg (Fig. 1a) and at 6 months from −2.2 to 2.8 kg (Fig. 1c). For FFM limits of agreement at 2 weeks ranged from −4.1 to 1.8 kg (Fig. 1b) and at 6 months from −3.4 to 2.1 kg (Fig. 1d).

Regression of the difference between the two methods against the mean of the two methods for FFM was significant at 2 weeks and 6 months, both $P < 0.005$ (Fig. 1b and 1d).

BMI and distribution of body water, measured as ECW/TBW, showed no association with FM or FFM at 2 weeks or 6 months (Supplementary Fig. 1a to h).

Ability of BIA to measure changes in body composition compared to DXA

The participants decreased in total weight and waist and hip circumference from 2 weeks pp to 6 months pp, along with a decrease in FM and FFM measured by both DXA and BIA, as well as a decrease in ECW/TBW (Table 2).

Table 2. Anthropometric variables and body composition variables from DXA and BIA examinations of the 94 women with a pre-pregnancy BMI of 25–35 kg/m^2 completing the baseline visit at 2 weeks postpartum and the follow-up visit at 6 months postpartum

	Baseline visit at 2 weeks postpartum		Follow-up visit at 6 months postpartum	Change baseline visit – follow-up visit	*P* forchange
	n	Mean (SD)	Mean (SD)	Mean (SD)	
**Weight (kg)**	94	87.0 (9.3)	82.3 (10.7)	−4.7 (4.8)	<0.001
**BMI (kg/m^2)**	94	30.6 (2.6)	29.0 (3.1)	−1.7 (1.7)	<0.001
**Waist circ. (cm)**	94	98.9 (8.4)	92.0 (9.1)	−6.9 (7.1)	<0.001
**Hip circ. (cm)**	94	115.9 (6.8)	111.1 (7.6)	−4.8 (4.2)	<0.001
**FM (kg)**					
DXA	94	36.5 (6.3)	33.6 (7.7)	−2.9 (4.1)	<0.001
BIA	94	35.7 (6.2)	33.3 (7.6)	−2.4 (4.1)	<0.001
Difference DXA-BIA	94	0.7 (1.4)	0.3 (1.3)	0.5 (1.1)	<0.001
**FFM (kg)**					
DXA	94	50.1 (5.1)	48.3 (5.0)	−1.8 (1.7)	<0.001
BIA	94	51.2 (4.6)	49.0 (4.5)	−2.3 (1.5)	<0.001
Difference DXA-BIA	94	−1.2 (1.5)	−0.7 (1.4)	−0.5 (1.0)	<0.001
**BIA variables**					
TBW (kg)	94	38.2 (3.5)	36.4 (3.5)	−1.8 (1.2)	<0.001
ECW (kg)	94	17.1 (1.6)	16.2 (1.6)	−0.9 (0.7)	<0.001
ECW:TBW (%)	94	44.8 (1.3)	44.4 (1.2)	−0.4 (1.0)	0.001

DXA, dual energy X-ray absorptiometry; BIA, bioelectrical impedance analysis; FM, fat mass; FFM, fat free mass; TBW, total body water; ECW, extracellular water

Pearson correlation for changes in FM and FFM between the two visits measured by DXA and BIA were 0.97 and 0.81 ($P < 0.001$), respectively.

Compared to DXA, BIA underestimated the change in FM by 0.5 (1.1) kg ($P < 0.001$) and overestimated the change in FFM by 0.5 (1.0) kg ($P < 0.001$), (Table 2).

Limits of agreement for changes in FM and FFM ranged from −1.7 to 2.6 kg and −2.5 to 1.5 kg, respectively (Fig. 1e and f).

For FM, there was no proportional bias between the methods, while for FFM there was a small proportional bias (Fig. 1f).

There was no proportional bias for change in BMI (Fig. 2a and b), however, regression of the difference in change between the two methods against change in ECW/TBW was significant for both FM and FFM, $P = 0.029$ and $P < 0.001$, respectively, (Fig. 2c and d).

Discussion

Similar to previous studies comparing BIA with DXA in cross-sectional samples (5–13), we also found in this sample of women with overweight or obesity that BIA underestimated the amount of FM and overestimated the amount of FFM compared to DXA, however, with narrower limits of agreement. This applied to measurements at both 2 weeks and 6 months pp. There was a proportional bias for overestimating FFM in the lower FFM range. On a group level, BIA underestimated the change in FM and overestimated the change in FFM from 2 weeks to 6 months pp, compared to DXA. The overestimation of change in FFM was larger in those women who increased their ECW relative to TBW.

Cross-sectional comparison at 2 weeks and 6 months

The difference between the methods in measuring FM and FFM was larger at 2 weeks pp (2.0–2.5%) compared to 6 months pp (0.7–1.5%). Similarly, limits of agreement were wider at 2 weeks pp (±2.7 – ±2.9) compared to 6 months pp (±2.5 – ±2.8). Thus, it seems that BIA has better precision in estimating both FM and FFM at 6 months pp. Considering the expected 'normalization' in hydration over time pp, this is not surprising (21). However, there was no proportional bias by the ratio of extracellular water to TBW at the two timepoints.

The limits of agreement seen here were more narrow, actually halved, compared to what we have shown in a previous study in a similar population of pp overweight and obese women in Sweden, using bioelectrical impedance spectroscopy (BIS) compared to DXA (8). The observed limits of agreement in this study are more in line with a study that used the same two devices with manufacturers' equations in pp women at 1 and 4 months pp (11), indicating that this eight-electrode BIA has high relative validity in comparison

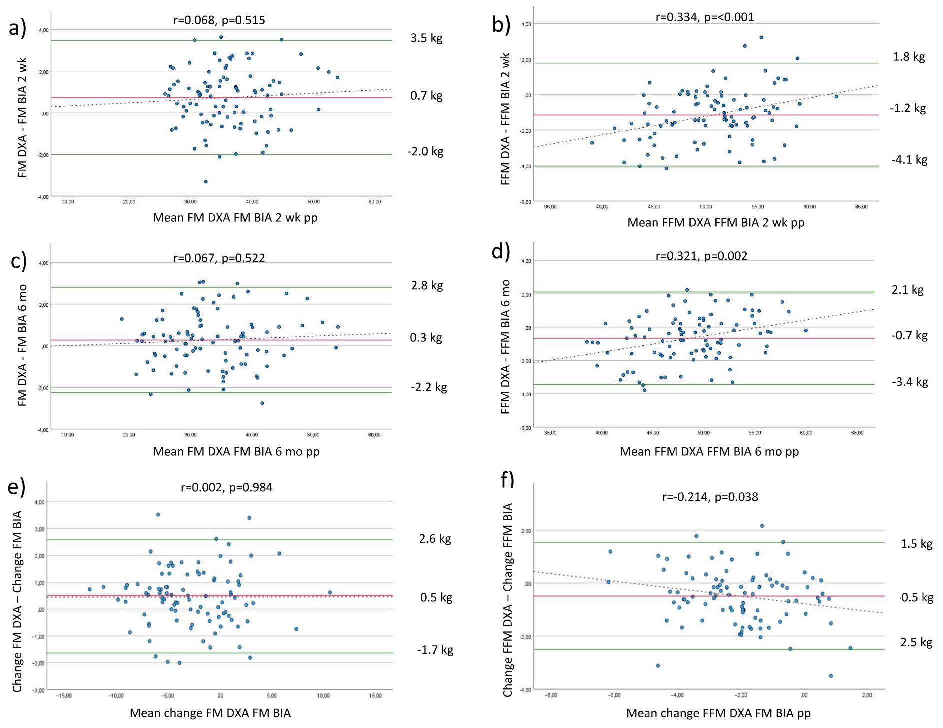

Fig. 1. Bland-Altman plots of cross-sectional comparison between DXA and BIA at 2 weeks (a, b) and 6 months (c, d) as well as longitudinal change between 2 weeks and 6 months (e, f) postpartum. DXA, dual energy X-ray absorptiometry; BIA, bioelectrical impedance analysis; FM, fat mass; FFM, fat free mass; pp, postpartum; wk, weeks; mo, months. y-axis shows reference method (DXA) minus test method (BIA).

to earlier generations of BIA devices. Although mean bias for FFM in pp women may be somewhat larger in comparison to studies using the same equipment in healthy men and women (9) and colorectal cancer patients (22), limits of agreement are generally more narrow. One likely explanation is the homogeneity of our population in terms of gender, age and BMI.

Measuring longitudinal changes in body composition

We found that BIA underestimated the change in FM by mean (SD) 0.5 (1.1) kg and overestimated the change in FFM by 0.5 (1.0) kg, which is similar to the results of Verdich et al. (23) who compared multifrequency BIA to Lunar iDXA during weigh loss. However, Bärebring et al. (24) found that single frequency BIA underestimated change in FFM during follow-up in colorectal patients. Differences between studies may be due to differences in weight change, population and type of BIA equipment.

The limits of agreement were approximately ±2 kg for longitudinal changes in both FM and FFM. This is about half of what Verdich et al. and Bärebring et al. found in their studies (23, 24). It is also much more narrow than we previously showed when comparing BIS to DXA (Lunar Prodigy) in a similar group of women (8). Thus, it seems that the Seca mBCA 515 has better agreement compared to other BIA devices, also when it comes to measuring longitudinal changes.

The ratio of ECW/TBW decreased significantly between the two timepoints and there was a highly significant proportional bias for measuring change in FM and FFM by change in water retention. Hopkinson et al. have shown that hydration of FFM in women 2 weeks pp is increased (0.75) compared to 'normal' hydration (0.73, typically assumed in 2-compartment models) and that hydration was higher in lactating (0.75) compared to non-lactating (0.74) women (25). These pregnancy-induced changes in hydration were no longer evident at 3 months pp, nor were there any differences between lactating and non-lactating women at that timepoint (21). Thus, although the manufacturers equation of this BIA device does not show any proportional bias with water retention in the cross-sectional comparison with DXA, the change in hydration seems to affect the agreement between BIA and DXA when measuring longitudinal change.

GE Healthcare Lunar iDXA has a precision error found to be small, less than 1% (26), also for the actual machine used in our study (19). It is important to notice that DXA also assumes a fixed hydration of 73% (27) while the four-compartment model, which is considered the gold standard for measuring body composition (28–30), does not assume a fixed hydration. Thus, DXA has some of the same limitations as BIA and may be affected by the

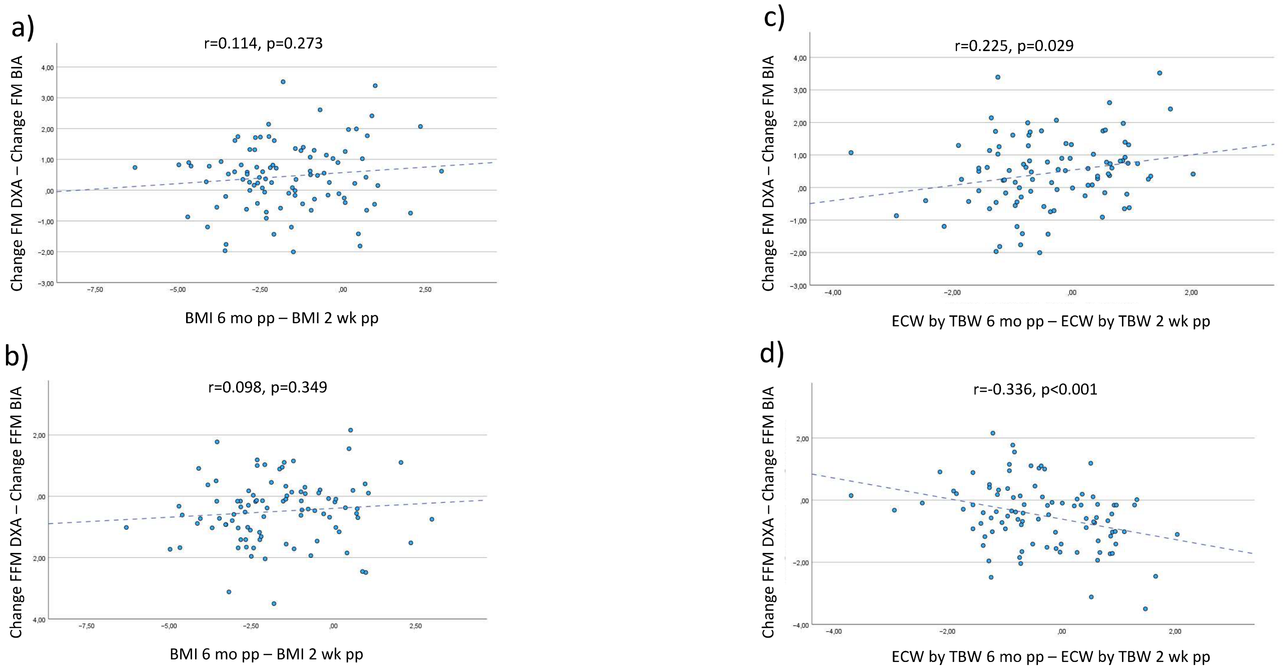

Fig. 2. Plots of the difference in change between FM and FFM by DXA – BIA and difference in BMI (a, b) as well as difference in ECW by TBW (c, d) between 2 weeks and 6 months postpartum. DXA, dual energy X-ray absorptiometry; BIA, bioelectrical impedance analysis; FM, fat mass; FFM, fat free mass; pp, postpartum; wk, weeks; mo, months. y-axis shows reference method (DXA) minus test method (BIA).

hydration status of our participants. Although it has been estimated that fat free tissue hydration between 68.2 and 78.2% does not significantly alter total percentage of fat (31), it has been shown that diet and training regimens that induce short-term changes in hydration can manipulate lean tissue mass results (32). The data on pp women from Butte et al. indicate that the composition of FFM in our participants are not affected by their pp or lactation status at 6 months pp as composition of FFM is expected to be normalized by 3 months pp (21). However, our results at 2 weeks pp may well be affected.

Although previous researchers have concluded that BIA is not a reliable method to track changes in body composition (13, 33), especially not in an overweight population (33), our results showed a good correlation between Seca mBCA 515 and DXA, with a degree of underestimation of changes in FM and overestimation of changes in FFM that for most purposes could be considered acceptable in a clinical setting. Garr Barry et al. (11) did also find the same BIA to be reliable for measuring FM and FFM in women at 1 and 4 months pp, both cross sectionally and longitudinally, in a similar, but smaller group of women, although weight stable. In addition, we have shown that this applies to women already 2 weeks pp and until 6 months pp, also during weight loss and without proportional bias for change in BMI, but with a proportional bias for change in hydration.

Limitations

The measurements in this comparison study were performed over a period of 4 years by several members of the project team and an interobserver variation cannot be excluded. However, training and standard operation procedures have been in place in order to minimize the variation.

The measurements were taken between 8 o'clock and 11 o'clock in the morning and the duration in an upright position may have affected BIA measurement (34). Neither intake of water (up to 200 mL) or time since last breastfeeding were recorded.

The light clothing was not standardized and was thus included in the calculations of FM and FFM made by BIA, but not by DXA.

It is unlikely that the diet intervention or BPI would affect the difference between the two methods, cross-sectionally or longitudinally, although this possibility cannot be excluded. Most women lose weight naturally from 2 weeks to 6 months pp and in addition half of the participants received weight loss treatment. Therefore, the results obtained here are mainly limited to a population undergoing weight loss pp.

Conclusion

At group level, BIA was a valid tool compared to DXA for assessment of FM and FFM in women with overweight

and obesity at 2 weeks and 6 months pp although the agreement improved with time pp. We also considered it valid for following changes in FM and FFM over time when fluid distribution is stable.

Acknowledgments

The authors would like to thank Ane Kjosås, Synne Risan Sævre, Tonje Bergsjø, Karoline Østereng Nilsen, Eline Hjellup Horne and Julie Glimsdal for participation in the data collection. They also thank the study participants for their time and efforts.

Conflict of interest and funding

The authors declared no conflict of interest. This study was funded by The Dam Foundation, via Norwegian Women's Public Health Association, The Throne-Holst Foundation for Nutrition Research, The Norwegian Research Council, the University of Oslo and Oslo University Hospital. The funders had no role in the design, analysis or writing of this article.

Authors' contribution

HKB and EAØ designed research, EW, EAØ, MF and HBH contributed to acquisition of data, EW and HKB analysed data. All authors contributed to the writing and approval of the final manuscript.

Data availability

The datasets generated during and/or analyzed during this study are not publicly available due to risk of re-identifying participants but are available from the corresponding author on reasonable request.

References

1. Lipsky LM, Strawderman MS, Olson CM. Maternal weight change between 1 and 2 years postpartum: the importance of 1 year weight retention. Obesity (Silver Spring) 2012; 20(7): 1496–502. doi: 10.1038/oby.2012.41
2. Williamson DF, Madans J, Pamuk E, Flegal KM, Kendrick JS, Serdula MK. A prospective study of childbearing and 10-year weight gain in US white women 25 to 45 years of age. Int J Obes Relat Metab Disord 1994; 18(8): 561–9.
3. Mullaney L, O'Higgins AC, Cawley S, Daly N, McCartney D, Turner MJ. Maternal weight trajectories between early pregnancy and four and nine months postpartum. Public Health 2016; 135: 144–6. doi: 10.1016/j.puhe.2016.02.017
4. Kyle UG, Bosaeus I, De Lorenzo AD, Deurenberg P, Elia M, Gomez JM, et al. Bioelectrical impedance analysis – part I: review of principles and methods. Clin Nutr 2004; 23(5): 1226–43. doi: 10.1016/j.clnu.2004.06.004
5. Sillanpaa E, Cheng S, Hakkinen K, Finni T, Walker S, Pesola A, et al. Body composition in 18- to 88-year-old adults – comparison of multifrequency bioimpedance and dual-energy X-ray absorptiometry. Obesity (Silver Spring) 2014; 22(1): 101–9. doi: 10.1002/oby.20583
6. Volgyi E, Tylavsky FA, Lyytikainen A, Suominen H, Alen M, Cheng S. Assessing body composition with DXA and bioimpedance: effects of obesity, physical activity, and age. Obesity (Silver Spring) 2008; 16(3): 700–5. doi: 10.1038/oby.2007.94
7. Wang JG, Zhang Y, Chen HE, Li Y, Cheng XG, Xu L, et al. Comparison of two bioelectrical impedance analysis devices with dual energy X-ray absorptiometry and magnetic resonance imaging in the estimation of body composition. J Strength Cond Res 2013; 27(1): 236–43. doi: 10.1519/JSC.0b013e31824f2040
8. Ellegard L, Bertz F, Winkvist A, Bosaeus I, Brekke HK. Body composition in overweight and obese women postpartum: bioimpedance methods validated by dual energy X-ray absorptiometry and doubly labeled water. Eur J Clin Nutr 2016; 70(10): 1181–8.
9. Day K, Kwok A, Evans A, Mata F, Verdejo-Garcia A, Hart K, et al. Comparison of a Bioelectrical impedance device against the reference method dual energy X-ray absorptiometry and anthropometry for the evaluation of body composition in adults. Nutrients. 2018; 10(10): 1469. doi: 10.3390/nu10101469.
10. Faria SL, Faria OP, Cardeal MD, Ito MK. Validation study of multi-frequency bioelectrical impedance with dual-energy X-ray absorptiometry among obese patients. Obes Surg 2014; 24(9): 1476–80. doi: 10.1007/s11695-014-1190-5
11. Garr Barry V, Martin SL, Chandler-Laney P, Carter EB, Worthington CS. A comparison of bioimpedance analysis vs. dual X-ray absorptiometry for body composition assessment in postpartum women and non-postpartum controls. Int J Environ Res Public Health. 2022;19(20):13636. doi: 10.3390/ijerph192013636
12. Neovius M, Hemmingsson E, Freyschuss B, Udden J. Bioelectrical impedance underestimates total and truncal fatness in abdominally obese women. Obesity (Silver Spring) 2006; 14(10): 1731–8. doi: 10.1038/oby.2006.199
13. Dona E, Olveira C, Palenque FJ, Porras N, Dorado A, Martin-Valero R, et al. Body composition measurement in bronchiectasis: comparison between bioelectrical impedance analysis, skinfold thickness measurement, and dual-energy X-ray absorptiometry before and after pulmonary rehabilitation. J Acad Nutr Diet 2018; 118(8): 1464–73. doi: 10.1016/j.jand.2018.01.013
14. Kyle UG, Bosaeus I, De Lorenzo AD, Deurenberg P, Elia M, Manuel Gomez J, et al. Bioelectrical impedance analysis-part II: utilization in clinical practice. Clin Nutr 2004; 23(6): 1430–53. doi: 10.1016/j.clnu.2004.09.012
15. Bosy-Westphal A, Schautz B, Later W, Kehayias JJ, Gallagher D, Muller MJ. What makes a BIA equation unique? Validity of eight-electrode multifrequency BIA to estimate body composition in a healthy adult population. Eur J Clin Nutr 2013; 67 Suppl 1: S14–21. doi: 10.1038/ejcn.2012.160
16. Jensen B, Braun W, Geisler C, Both M, Kluckmann K, Muller MJ, et al. Limitations of fat-free mass for the assessment of muscle mass in obesity. Obes Facts 2019; 12(3): 307–15. doi: 10.1159/000499607
17. Mottelson MN, Lundsgaard CC, Moller S. Mechanisms in fluid retention – towards a mutual concept. Clin Physiol Funct Imaging. 2020; 40(2): 67–75. doi: 10.1111/cpf.12615
18. Alavi DH, Henriksen HB, Lauritzen PM, Kvaerner AS, Sakinis T, Langleite TM, et al. Quantification of adipose tissues by dual-energy X-ray absorptiometry and computed tomography in colorectal cancer patients. Clin Nutr ESPEN 2021; 43: 360–8. doi: 10.1016/j.clnesp.2021.03.022
19. Henriksen HB, Alavi DH, Blomhoff R. Precision of lunar dual-energy X-ray Absorptiometry (iDXA) in measuring body

composition among colorectal cancer patients and healthy subjects. Clin Nutr ESPEN 2021; 44: 316–23. doi: 10.1016/j.clnesp.2021.05.025
20. Hangartner TN, Warner S, Braillon P, Jankowski L, Shepherd J. The Official Positions of the International Society for Clinical Densitometry: acquisition of dual-energy X-ray absorptiometry body composition and considerations regarding analysis and repeatability of measures. J Clin Densitom 2013; 16(4): 520–36. doi: 10.1016/j.jocd.2013.08.007
21. Butte NF, Hopkinson JM, Ellis KJ, Wong WW, Smith EO. Changes in fat-free mass and fat mass in postpartum women: a comparison of body composition models. Int J Obes Relat Metab Disord 1997; 21(10): 874–80. doi: 10.1038/sj.ijo.0800482
22. Raeder H, Kvaerner AS, Henriksen C, Florholmen G, Henriksen HB, Bohn SK, et al. Validity of bioelectrical impedance analysis in estimation of fat-free mass in colorectal cancer patients. Clin Nutr 2018; 37(1): 292–300. doi: 10.1016/j.clnu.2016.12.028
23. Verdich C, Barbe P, Petersen M, Grau K, Ward L, Macdonald I, et al. Changes in body composition during weight loss in obese subjects in the NUGENOB study: comparison of bioelectrical impedance vs. dual-energy X-ray absorptiometry. Diabetes Metab 2011; 37(3): 222–9. doi: 10.1016/j.diabet.2010.10.007
24. Barebring L, Kvaerner AS, Skotnes M, Henriksen HB, Skjetne AJ, Henriksen C, et al. Use of bioelectrical impedance analysis to monitor changes in fat-free mass during recovery from colorectal cancer- a validation study. Clin Nutr ESPEN 2020; 40: 201–7. doi: 10.1016/j.clnesp.2020.09.021
25. Hopkinson JM, Butte NF, Ellis KJ, Wong WW, Puyau MR, Smith EO. Body fat estimation in late pregnancy and early postpartum: comparison of two-, three-, and four-component models. Am J Clin Nutr 1997; 65(2): 432–8. doi: 10.1093/ajcn/65.2.432
26. Rothney MP, Martin FP, Xia Y, Beaumont M, Davis C, Ergun D, et al. Precision of GE Lunar iDXA for the measurement of total and regional body composition in nonobese adults. J Clin Densitom 2012; 15(4): 399–404. doi: 10.1016/j.jocd.2012.02.009
27. Laskey MA. Dual-energy X-ray absorptiometry and body composition. Nutrition 1996; 12(1): 45–51. doi: 10.1016/0899-9007(95)00017-8
28. Ng BK, Liu YE, Wang W, Kelly TL, Wilson KE, Schoeller DA, et al. Validation of rapid 4-component body composition assessment with the use of dual-energy X-ray absorptiometry and bioelectrical impedance analysis. Am J Clin Nutr 2018; 108(4): 708–15. doi: 10.1093/ajcn/nqy158
29. Williams JE, Wells JC, Wilson CM, Haroun D, Lucas A, Fewtrell MS. Evaluation of Lunar Prodigy dual-energy X-ray absorptiometry for assessing body composition in healthy persons and patients by comparison with the criterion 4-component model. Am J Clin Nutr 2006; 83(5): 1047–54. doi: 10.1093/ajcn/83.5.1047
30. Minderico CS, Silva AM, Keller K, Branco TL, Martins SS, Palmeira AL, et al. Usefulness of different techniques for measuring body composition changes during weight loss in overweight and obese women. Br J Nutr 2008; 99(2): 432–41. doi: 10.1017/S0007114507815789
31. Kelly TL, Berger N, Richardson TL. DXA body composition: theory and practice. Appl Radiat Isot 1998; 49(5–6): 511–3. doi: 10.1016/S0969-8043(97)00226-1
32. Toomey CM, McCormack WG, Jakeman P. The effect of hydration status on the measurement of lean tissue mass by dual-energy X-ray absorptiometry. Eur J Appl Physiol 2017; 117(3): 567–74. doi: 10.1007/s00421-017-3552-x
33. Thivel D, Verney J, Miguet M, Masurier J, Cardenoux C, Lambert C, et al. The accuracy of bioelectrical impedance to track body composition changes depends on the degree of obesity in adolescents with obesity. Nutr Res 2018; 54: 60–8. doi: 10.1016/j.nutres.2018.04.001
34. Thurlow S, Taylor-Covill G, Sahota P, Oldroyd B, Hind K. Effects of procedure, upright equilibrium time, sex and BMI on the precision of body fluid measurements using bioelectrical impedance analysis. Eur J Clin Nutr 2018; 72(1): 148–53. doi: 10.1038/ejcn.2017.110

***Hilde K. Brekke**
Department of Nutrition
Institute of Basic Medical Sciences
University of Oslo
PO 1046 Blindern
0317 Oslo
Norway
Email: h.k.brekke@medisin.uio.no
